# Effects of COVID-19 virus-like particles on the behavioral and cognitive performance of human apolipoprotein E targeted replacement mice

**DOI:** 10.3389/fimmu.2024.1473366

**Published:** 2024-11-26

**Authors:** Abigail O’Niel, Alexandra Pederson, Elizabeth Saltontall, Kayla Nguyen, Monzerrat Pantoja, Mitali Chaudhari, Phoebe Sandholm, Eric Yoon, Henry F. Harrison, Sydney Boutros, Alec J. Hirsch, Jacob Raber

**Affiliations:** ^1^ Department of Behavioral Neuroscience, Oregon Health & Science University, Portland, OR, United States; ^2^ Vaccine and Gene Therapy Center, ONPRC, Oregon Health & Science University, Portland, OR, United States; ^3^ Department of Neurology, Oregon Health & Science University, Portland, OR, United States; ^4^ Department of Radiation Medicine, Oregon Health & Science University, Portland, OR, United States; ^5^ Division of Neuroscience, Oregon National Primate Research Center (ONPRC), Oregon Health & Science University, Portland, OR, United States

**Keywords:** COVID-19, virus-like particles, apolipoprotein E, behavioral testing, cognitive testing

## Abstract

**Introduction:**

The effects of viral infections might be apolipoprotein E (apoE) isoform-dependent. In humans, there are three major apoE isoforms, E2, E3, and E4. E4 is associated with the enhanced entry of several viruses into the brain and their disease progression. A concern of infection by severe acute respiratory syndrome coronavirus 2 (SARS-CoV-2) is the development of post-acute COVID-19 syndrome, also known as long COVID. Genetic risk factors for developing long COVID were reported.

**Methods:**

In this study, we used virus-like particles (VLPs) that include expression of the SARS-CoV-2 nucleocapsid (N), membrane (M), and envelope (E) structural proteins together with S. In the current study, we used human E2, E3, and E4 targeted replacement mice to assess whether these VLPs affect body weight, behavioral and cognitive performance, and circadian body temperatures. Using VLPs allow working outside an ABSL-3 facility.

**Results:**

The effects of VLPs on some behavioral measures were apoE isoform-dependent, with the E2 mice being more affected than E3 or E4 mice. The overall decreased activity in the open field containing objects in week 2 indicate that VLPs can also reduce activity levels in an apoE isoform-independent fashion.

**Discussion:**

The results of the current study indicate that even in the absence of viral replication, detrimental effects of VLPs on behavioral measures and circadian body temperatures are seen.

## 1 Introduction

The effects of viral infections might be apolipoprotein E (apoE) isoform-dependent. E4 is associated with enhanced entry of several viruses into the brain and their disease progression, including that of human immunodeficiency virus-1 (HIV) (1), herpes simplex virus-1, hepatitis C virus, hepatitis E virus, varicella zoster virus, Epstein–Barr virus, malaria, *Listeria monocytogenes* (LM), and *Klebsiella pneumoniae* (2). The impact of COVID-19 might also be apoE isoform-dependent. E4 modifies the associations of polymorphisms in angiotensin-converting enzyme (ACE), which plays a key role in COVID-19 (3), with neuropsychiatric syndromes in Alzheimer’s disease (AD) (4). In addition, the *APOE* genotype is associated with survival in patients infected with COVID-19 (5); compared to E3 homozygous patients, E4 homozygous patients showed poorer survival. E2 homozygous patients showed a trend towards lower survival than E3 homozygous patients, but this did not reach significance, which might be related to the lower occurrence of E2 than E4 in the population. E4 is also associated with severe COVID-19 with more prevalent microhemorrhages in intensive care patients (6). Consistent with human studies, in human apoE mice, E2 and E4 mice showed worse survival than E3 mice following infection with COVID-19 (5).

Among the concerns of infection by severe acute respiratory syndrome coronavirus 2 (SARS-CoV-2) is the development of post-acute COVID-19 syndrome, also known as long COVID. Many of the symptoms of long COVID, including fatigue, myalgia, learning and memory impairments, anxiety, and a post-traumatic stress disorder-like condition, are likely mediated through the central nervous system (CNS) (7) as well as PMC9537254. Pre-existing psychiatric conditions might increase risk to develop long COVID (8). Genetic risk factors for developing long COVID were reported (9). There might be an overlap between risk to develop long COVID and risk to develop age-related neurodegenerative conditions such as AD (10). apoE plays a role in cholesterol metabolism and neuronal repair after injury (11). Compared to apoE3 (E3), apoE4 (E4) is a risk factor for developing cardiovascular disease and AD (12–14), while apoE2 (E2) provides relative protections against developing AD (15). However, in adverse environments, E4 might provide relative protection (16).

COVID-19 is a betacoronavirus (17), which possesses a large (26–32 kb) positive-sense RNA genome that interacts with the nucleocapsid (N) protein to form the ribonucleoprotein core of the virion, encased in the viral envelope and shaped by the membrane (M) protein. The envelope (E) protein forms an ion channel that is required for the virulence of SARS- and MERS-CoVs and is also thought to close the virion during budding (18). The spike (S) protein is arranged in trimers on the virion surface and mediates viral entry into the host cell. The majority of the antibody response during coronavirus infection is directed against the N and S proteins, with virus neutralization and protection of the host being mediated primarily by anti-S antibodies (19). The N, M, and E proteins of SARS-CoV-2, or minimally N and M, are sufficient for efficient release of virus-like particles (VLPs) into the culture medium of transfected cells (20). The S protein, which can be incorporated into VLPs as well binds to humanACE2 on the host cell.

Dr. Sullivan developed targeted replacement (TR) E2 (21), E3 (22), and E4 (23) mice that express human apoE under control of the mouse apoE promoter on a C57BL/6J background. In the current study, we used these mice to assess whether VLPs affect body weight, behavioral and cognitive performance, and circadian body temperatures in E2, E3, and E4 TR mice. As markers of immune response were elevated in COVID-19-infected mice and exposed patients, we also assessed hippocampal mRNA levels of tumor necrosis factor (TNF)-α, interleukin (IL)-4, interferon (IFN)-γ, and C-C motif chemokine 11 (CCL11 or Eotaxin). Expression of TNF-α was reported to be induced in the brains of SARS-CoV-2-infected mice at 7 dpi, but not at 7 weeks, while CCL11 levels were induced long term (24). IL-4 levels are associated with COVID-19 severity (25). Low levels of IFN-γ were suggested as a risk factor for hospitalization following exposure to COVID-19 and IFN-γ was shown to be associated with recovery following COVID-19 exposure (26, 27).

## 2 Materials and methods

### 2.1 Mice

TR E2 (21), E3 (22), and E4 (23) mice [*n* = 51 (10.51 ± 0.12 months of age); E2: *n* =15 (10.65 ± 0.22 months of age; *n* = 6 males and *n* = 9 females); E3: *n* = 17 (10.47 ± 0.24 months of age; *n* = 8 males and *n* = 9 females); E4: *n* = 19 (10.43 ± 0.19 months of age; *n* = 10 males and *n* = 9 females)] expressing human apoE under control of the mouse apoE promoter on a C57BL/6J background were used in this study. Homozygous breeding of the mice was used to generate the experimental mice for this study. Throughout testing, all the mice were singly housed. Animals were maintained on a 12:00 h light/dark schedule (lights on at 06:00). Laboratory chow (PicoLab Rodent diet 20, # 5053; PMI Nutrition International, St. Louis, MO, USA) and water were provided *ad libitum*. Behavioral testing took place during the light cycle. All procedures complied with the National Institutes of Health Guide for the Care and Use of Laboratory Animals and with IACUC approval at Oregon Health & Sciences University. Experimenters were blinded to the genotype, sex, and treatment of the mice.

### 2.2 Implantation of temperature sensors

TS100 millimeter-scale (7.5 × 7.5 × 4.2 mm) CubiSensTM wireless sensors (CubeWorks, Ann Arbor, MI), packaged in bio-compatible epoxy and coated with parylene, were implanted in the abdomen for accurate, real-time temperature measurement. The TS100 is capable of transmitting up to 100 m in distance, lasts up to 2 years in sensing operation, and allows measuring circadian body temperature in individual mice. The sensors were sterilized using the Cidex solution (CubeWorks, Ann Arbor, MI). A heating pad and bead sterilizer were used for the surgeries.

For the surgery, the mice were anesthetized with isoflurane (4% for induction of the anesthesia and 1%–3% for maintenance of the anesthesia). Lidocaine (7 mg/kg of 0.5%) was injected subcutaneously around the incision site, immediately prior to the aseptic preparation of the abdomen. To close the abdominal cavity, 4-0 undyed, unbraided, monofilament sutures were used. To close the skin, 9-mm AUTOCLIP stainless steel clips were used. For pain control, meloxicam (10 mg/kg) was administered orally prior to the induction of anesthesia and every 24 h for two additional days. The mice were treated and behaviorally tested starting 2 weeks after the surgeries. Body temperatures were acquired and analyzed for the first week of behavioral testing.

### 2.3 Generation of SARS-CoV-2 VLPs

**Figure 1:**
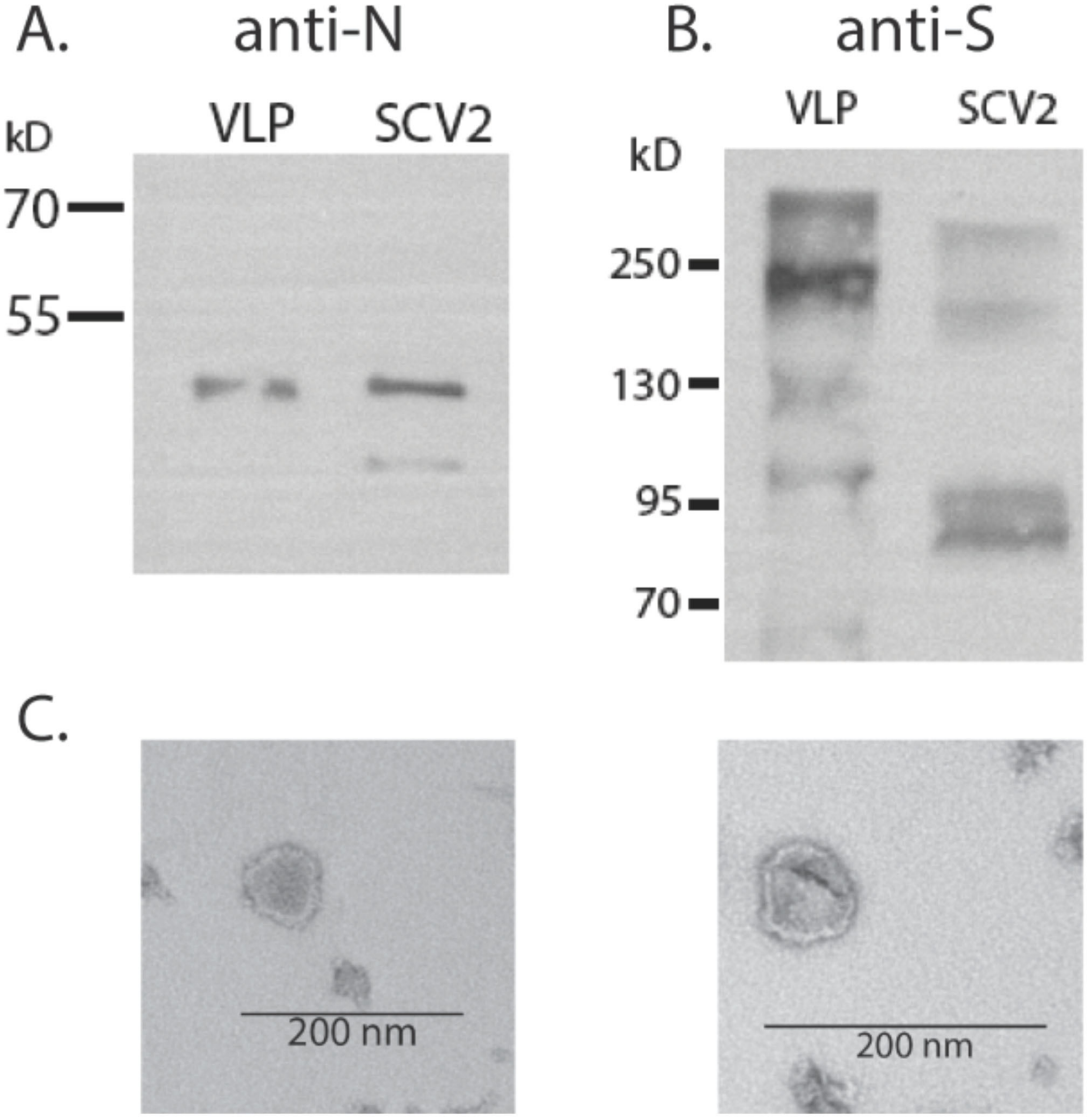
Production of SARS-CoV-2 VLPs. **(A)** Western blot detection of N protein in VLPs or SARS-CoV-2 virus (3×10^5^ focus forming units). **(B)** Detection of S in VLP and virus. **(C)** Electron micrograph of VLP prep.

The VLPs were generated to express the SARS-CoV-2 nucleocapsid (N), membrane (M), and envelope (E) structural proteins together with S, allowing us to perform SARS-CoV-2-related studies without replicating virus and outside a BSL-3 facility. Plasmid expression vectors encoding each of the SARS-CoV-2 N, M, E, and S proteins were constructed by standard cloning methods, using synthesized codon-optimized sequences. M, E, N, and S plasmids were transfected at a ratio of 5 µg:1 µg:5 µg:1 µg into suspension-grown Expi293F (25 mL at 3×10^6^ cells/mL) cells using ExpiFectamine reagent (ThermoFisher). Cells were allowed to grow for 4–6 days post-transfection before harvesting. Cells were separated from culture supernatant by centrifugation at 2,000 rpm for 15 min, and culture supernatant was passed through a 0.45-µm filter. VLPs were further concentrated and purified by ultracentrifugation through a 20% sorbitol cushion at 30,000 rpm for 2 h. The pellet was resuspended in 1/100 original volume of phosphate-buffered saline (PBS). Viral proteins S and N were detected by Western blotting (**Figures 1A, B**), and the VLP structure was assessed by transmission electron microscopy of negatively stained samples (**Figure 1C**). Total protein content will be assessed by BCA assay.

### 2.4 Treatments

For two subsequent weeks, mice were injected daily (weekdays 1–5), each morning 1 h prior to the first behavioral test of that day, with virus-like particles (VLPs) (1 μg/mouse) or vehicle, intraperitoneally, in a volume of 100 μL. The dose was selected based on a preliminary study showing that that dose showed a robust threefold increase in plasma corticosterone levels 1 h following i.p. injection, while a lower dose of 0.3 μg did not.

### 2.5 Body weights

Body weights were taken prior to the surgery and at the time of the grip strength tests, on day 4 of weeks 1 and 2. The body weight ratio for both weeks was calculated as outcome measure and defined as: (body weight at the time of the grip strength test − body weight prior to the surgery)/(body weight prior to the surgery).

### 2.6 Behavioral testing

**Figure 2:**
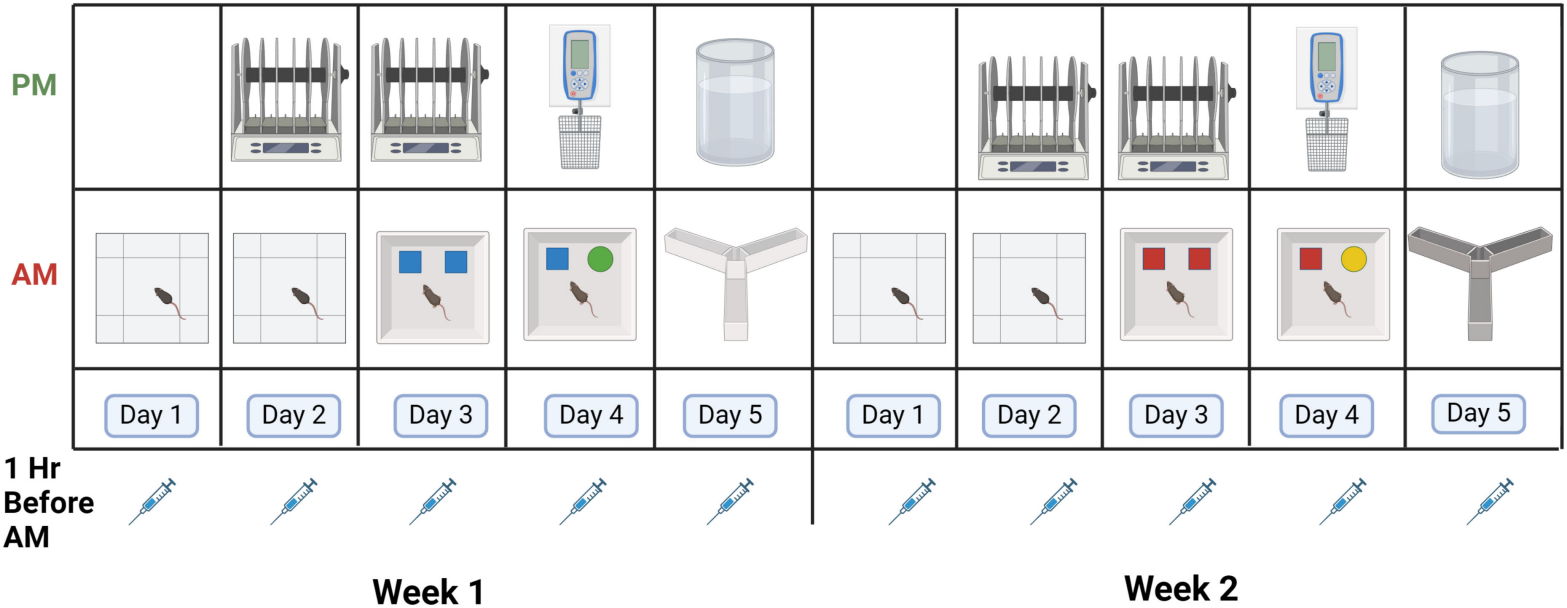
Schedule of behavioral testing. Mice were injected 5 days per week in weeks 1 and 2 with VLPs or vehicle. For details, see text.

Mice were behaviorally tested as follows (**Figure 2**). In the morning of days 1 and 2 of both weeks, mice were tested for measures of activity, measures of anxiety, and spatial habituation in the open field. In the morning of days 3 and 4 of both weeks, the mice were tested for object recognition. In the morning of day 5 of both weeks, mice were tested for spontaneous alternation in the Y maze. In the afternoon of days 2 and 3 of both weeks, the mice were tested for sensorimotor function on the rotarod. In the afternoon of day 4 of both weeks, the mice were tested for grip strength. In the afternoon of day 5 of both weeks, the mice were tested for depressive-like behavior in the forced swim test. The behavioral tests were performed as described below.

### 2.7 Open field and novel object recognition

The mice were put in an open field enclosure (16 × 16 inches, Kinder Scientific, Poway, CA) for 10 min on two subsequent days. On day 3, the open field contained two identical objects for a 15-min trial. The next day, one object was replaced with a novel object for a 15-min trial. Between trial, the arenas and objects were cleaned with 0.5% acetic acid. Interaction within a 2-cm proximity with the object was coded as object exploration by hand scoring videos acquired with Noldus Ethovision software (version 17, Wageningen, The Netherlands). A discrimination index was defined as the time spent exploring the familiar object subtracted from the time exploring the novel object, and dividing the resulting number by the total time spent exploring both objects. A positive discrimination index indicates a preferential exploration of the novel object. A negative discrimination index indicated a preferential exploration of the familiar object. Different objects were used during the first and second week of testing.

The outcome measures in the open field analyzed were as follows (1): distance moved in the open field in the absence and presence of objects, an activity measure (2); the difference in the distance moved in the open field over days, habituation to the open field, a cognitive measure (3); time spent in the center of the open field, an anxiety measure; and (4) the discrimination index, a cognitive measure.

### 2.8 Grip strength

A Harvard Apparatus (Holliston, MA) grip strength meter for mice was positioned horizontally. The mice were allowed to grasp the metal grid and pulled backwards in the horizontal plane. The force applied to the grid was recorded as the peak tension. Three measurements were conducted at 1-min intervals (28). The peak grip strength for each mouse was recorded. In addition, we calculated the relative grip strength as the ratio of grip strength to body weight, as previously described (29). The outcome measures in the grip strength test were the peak grip strength and the ratio of grip strength to body weight.

### 2.9 Y maze

Activity levels and hippocampus-dependent spontaneous alternations were assessed in week 1 in the Y maze from Harvard Apparatus (Panlab, Holliston, MA, United States). This Y maze is smaller and distinct [raised sides and made of non-reflective opaque gray plastic (30 cm × 6 cm × 15 cm)] from the one used during the second week of testing. For the second week of testing, we used the Y-shaped maze from O’ Hara & Co., Ltd. (Tokyo, Japan) that had raised sides (3.8 cm bottom width, 12.55 cm top width, and 12.55 cm height) with plastic, opaque gray arms (37.98 cm length). The maze was cleaned with 0.5% acetic acid between trials. Performance was assessed during a 5-min trial. Performance was recorded using the Noldus Ethovision software and hand scoring was used to assess the number of arm entries and the percent spontaneous alternations. The outcome measures in the Y maze were total arm entries, an activity measure, and percent spontaneous alternations, a cognitive measure.

### 2.10 Rotarod

The rotarod test (rod diameter: 3 cm, elevated: 45 cm; Rotamex-5, Columbus Instruments, Columbus, OH, USA) was used to assess sensorimotor function. The rotation speed started at 5 rpm and accelerated 1.0 rpm every 3 s. Fall latency (s) was recorded. For both weeks of testing, mice received three subsequent trials on two subsequent days. The outcome measure in the rotarod test used was the mean fall latency of each mouse for each day.

### 2.11 Forced swim test

To assess depressive-like behavior, mice were placed for 6 min in a container with water (water height: 15 cm; container diameter: 16–20 cm; 25°C) not allowing the mouse’s tail to touch the bottom. Immobility, defined as cessation of limb movements except for minor involuntary movements of the hind limbs or those movements necessary to stay afloat, was scored manually by an observer blinded to genotype and test history using a sampling technique every 5 s during the trial. The data are expressed as the percentage of immobility (number of immobility observations divided by the total number of observations) during the last 4 min (=48 observations) of the test, as previously described (30).

### 2.12 Hippocampal cytokine mRNA expression

Following the forced sim test, the mice were euthanized by cervical dislocation. The brain was quickly removed and the hippocampi were dissected in ice-cold PBS and stored at −80°C for analysis. RNA isolated from hippocampi was analyzed by qRT-PCR for expression of the inflammatory mediators TNF-α, IFN-γ, IL-4, and CCL11. RNA was extracted using TRIzol reagent (Invitrogen) according to the manufacturer’s protocol. Relative expression of cytokines was determined by qRT-PCR using gene-specific primer-probe sets (ThermoFisher) and normalized to β-actin mRNA expression using the ΔΔCt method (31).

### 2.13 Statistical analyses

All behavioral data are reported as mean ± standard error of the mean and were analyzed using SPSS v.22 (IBM, Armonk, NY, USA) or GraphPad v.8 (La Jolla, CA, USA) software. Genotype and treatment were included as factors in analysis of variance (ANOVA), and sex was used as a covariate. In case there were statistical genotype interactions, genotypes were analyzed separately, as indicated. There were no statistical treatment × sex interactions. When sex or an interaction with sex was not significant, we dropped sex as a covariate and reran the analysis. Repeated measures were used when appropriate. As the E2, E3, and E4 mice were of similar age, age was not included as part of the analysis. The 2 weeks of open field testing and the 2 weeks of testing behavioral performance in the open field containing objects were analyzed separately using day as the repeated measure. Because of the strong practice effects in the rotarod tests, we analyzed the four subsequent days of rotarod testing over the 2 weeks using a repeated measures ANOVA. For the other behavioral tests, week was used as the repeated measure in the ANOVA. For the circadian data, based on the pattern of the data, the light and dark periods for the 5 days were analyzed as separate analyses, with the mean body temperature in the light or dark period of each day as the repeated measure. Based on the three-way treatment × genotype × treatment interaction revealed, we next analyzed the VLP- and vehicle-treated genotype-matched group separately and finally performed an analysis of each light and dark period separately, with the hour as the repeated measure. Statistical significance was considered as *p* < 0.05. When sphericity was violated (Mauchly’s test), Greenhouse–Geisser corrections were used. Mice were tested in separate cohorts, each containing mice of all experimental groups. All researchers were blinded to genotype and treatment, and the code was only broken after the data were analyzed.

To determine the relationships between behavioral performance measures on the different tests in individual mice, a principal components analysis (PCA) was performed. The behavioral measures used for this analysis are indicated in **Table 1**. The PCA was performed using SPSS software and using the varimax rotated matrix. Factors with eigenvalues > 1 were considered significant.

**Table 1:** Behavioral measures used in the PCA.

Abbreviation	Behavioral Measure
TDOFD1w1	Total distance moved on the first day in the open field in week 1.
TDOFD2w1	Total distance moved on the second day in the open field in week 1.
TDOFD3w1	Total distance moved on the third day in the open field in week 1.
TDOFD4w1	Total distance moved on the fourth day in the open field in week 1.
TDOFD1w2	Total distance moved on the first day in the open field in week 2.
TDOFD2w2	Total distance moved on the second day in the open field in week 2.
TDOFD3w2	Total distance moved on the third day in the open field in week 2.
TDOFD4w2	Total distance moved on the fourth day in the open field in week 2.
CDOFD1w1	Distance moved in the center of the open field on the first day in the open field in week 1.
CDOFD2w1	Distance moved in the center of the open field on the second day in the open field in week 1.
CDOFD3w1	Distance moved in the center of the open field on the third day in the open field in week 1.
CDOFD4w1	Distance moved in the center of the open field on the fourth day in the open field in week 1.
CDOFD1w2	Distance moved in the center of the open field on the first day in the open field in week 2.
CDOFD2w2	Distance moved in the center of the open field on the second day in the open field in week 2.
CDOFD3w2	Distance moved in the center of the open field on the third day in the open field in week 2.
CDOFD4w2	Distance moved in the center of the open field on the fourth day in the open field in week 2.
DIw1	Discrimination Index in the object recognition test in week 1.
DIw2	Discrimination Index in the object recognition test in week 2.
Immobility FSTw1	Percent immobility in the Forced Swim Test in week 1.
Immobility FSTw2	Percent immobility in the Forced Swim Test in week 2.
Entries Ymazew1	Number of entries in the Y maze in week 1.
SpontAlternationw1	Spontaneous Alternations in the Y maze in week 1.
Entries Ymazew2	Number of entries in the Y maze in week 2.
SpontAlternationw2	Spontaneous Alternations in the Y maze in week 1.
GripStrengthw1	Grip strength in the grip strength test in week 1.
GripStrengthw2	Grip strength in the grip strength test in week 2.
RRD1w1	Fall latency on the first day of the rotarod test in week 1.
RRD2w1	Fall latency on the second day of the rotarod test in week 1.
RRD1w2	Fall latency on the first day of the rotarod test in week 2.
RRD2w2	Fall latency on the second day of the rotarod test in week 2.
BWRatiow1	Body weight ratio in week 1.
BWRatiow2	Body weight ratio in week 2.

Hippocampal cytokine mRNA expression levels were analyzed in each genotype using *t*-tests.

## 3 Results

### 3.1 Body weights/

**Figure 3:**
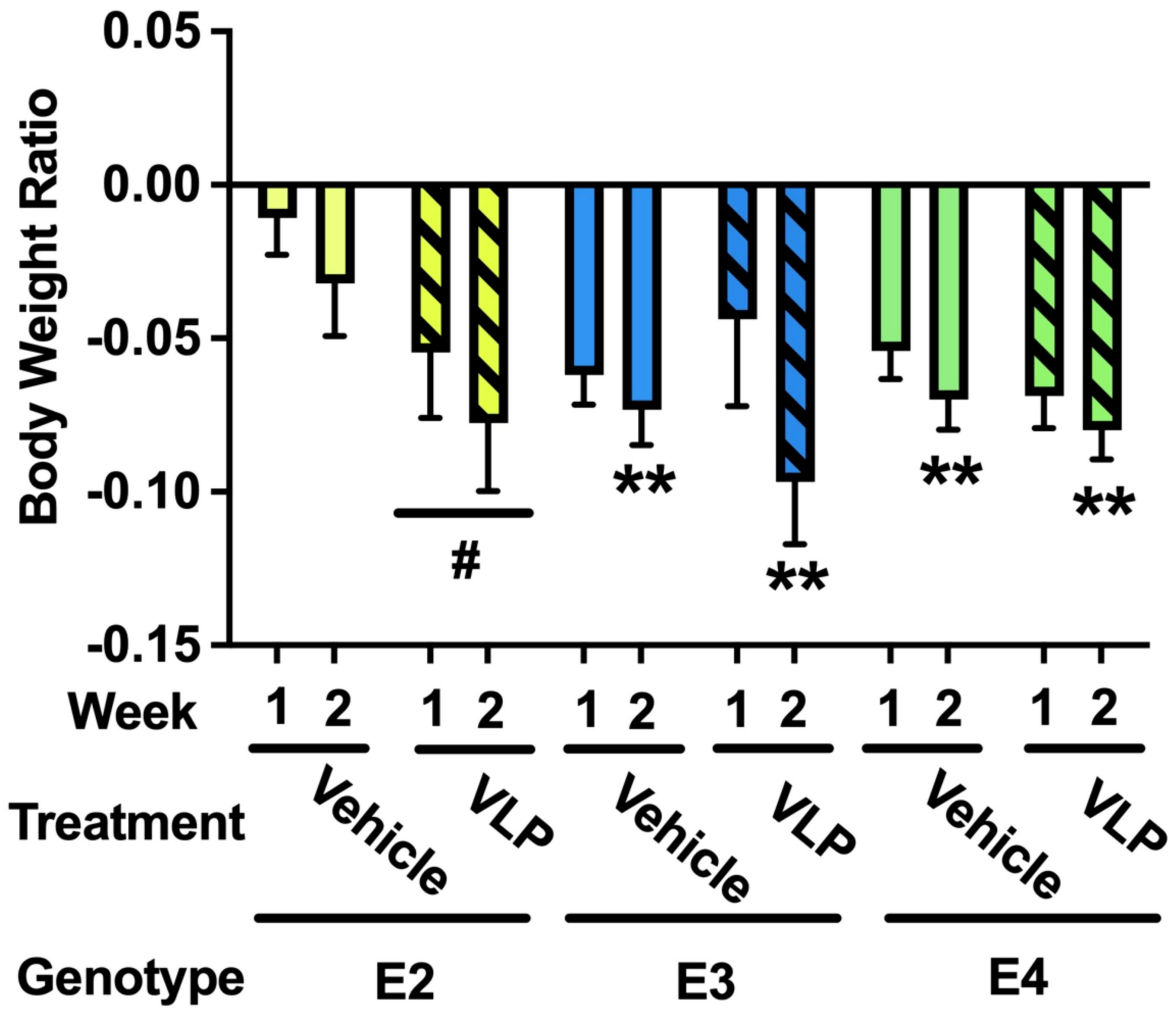
Body weight ratios in VLP- and vehicle-treated E2, E3, and E4 mice. In E2 mice, there was a trend towards an effect of treatment with a trend towards a lower body weight ratio in VLP- than saline-treated mice. ^#^ *p* = 0.0676. In E3 and E4 mice, there was only an effect of week. **p < 0.01.

For body weights, there was an effect of week [*F* (1,44) = 10.638, *p* = 0.002], with a lower body weight ratio in week 2 than in week 1, and there was a trend towards a week × genotype × treatment interaction [*F*(2,44) = 2.810, *p* = 0.071] (**Figure 3**). In E2 mice, there was a trend towards an effect of treatment [*F*(1,13) = 3.976, *p* = 0.0676], with a trend towards a lower body weight ratio in VLP- than vehicle-treated E2 mice. In E3 mice, there was only an effect of week [*F*(1,15) = 8.858, *p* = 0.0094], with a lower body weight ratio in week 2 than in week 1. Similarly, in E4 mice, there was only an effect of week [*F*(1,17) = 12.85, *p* = 0.0023], with a lower body weight ratio in week 2 than in week 1.

### 3.2 Open field and novel object recognition

**Figure 4:**
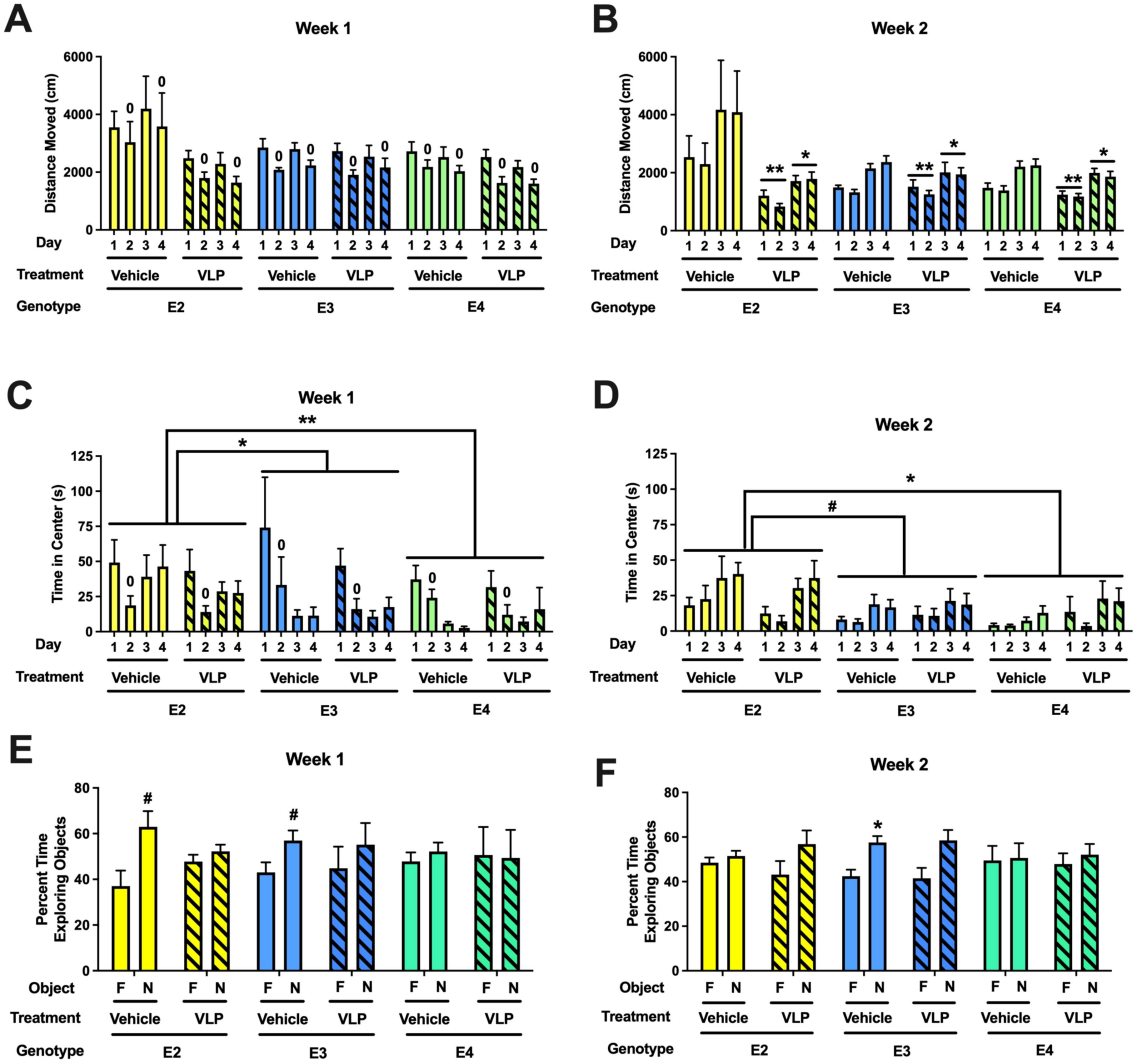
**(A)** Behavioral performance in the open field and novel object recognition of VLP- and vehicle-treated E2, E3, and E4 mice. Activity levels in the open field without (days 1 and 2) and with objects (days 3 and 4) during week 1. There was overall spatial habituation to the open field, with lower activity levels on day 2 than day 1. ^0^ *p* < 0.001 versus day 1. **(B)** Activity levels in the open field without (days 1 and 2) and with objects (days 3 and 4) during week 2. VLP-treated mice moved less than vehicle-treated mice. **p = 0.003 versus vehicle-treated mice on days 1 and 2, *p = 0.038 versus vehicle-treated mice on days 3 and 4. **(C)** Time spent in the center of the open field without (days 1 and 2) and with objects (days 3 and 4) during week 1. Mice spent less time in the center of the open field on day 2 than day 1. ^0^ *p* < 0.001. In addition, E2 mice spent more time in the center of the open field than E3 or E4 mice. *p = 0.0155, **p = 0.0024, Tukey’s. **(D)** Time spent in the center of the open field without (days 1 and 2) and with objects (days 3 and 4) during week 2. E2 mice spent more time in the center of the open field than E4 mice and there was a trend towards E2 mice spending more time in the center of the open field than E3 mice. *p = 0.0333, ^#^ *p* = 0.056, Tukey’s. In the object recognition test, the time spent exploring the novel (N) and familiar **(F)** objects are analyzed for each group. **(E)** During week 1, vehicle-treated E2 (^#^ *p* = 0.0531, paired *t*-test) and E3 mice (#*p* = 0.0783, paired *t*-test) showed a trend towards exploring the novel object more than the familiar one. **(F)** During week 2, vehicle-treated E3 mice spent more time exploring the novel than the familiar object. *p = 0.0176, paired *t*-test.

When the activity levels in the open field during week 1 were analyzed, there was an effect of day, with lower activity levels on day 2 than day 1 [*F*(1,44) = 67.636, *p* < 0.001] (**Figure 4A**). In addition, there was a trend towards an effect of treatment [*F*(1,44) = 3.057, *p* = 0.087]. When the activity level in the open field containing objects (days 3 and 4) during week 1 were analyzed, there was an effect of day [*F*(1,44) = 41.925, *p* < 0.001] (**Figure 4A**).

When the activity levels in the open field during week 2 were analyzed, there was an effect of treatment [*F*(1,44) = 10.183, *p* = 0.003] and a genotype × treatment interaction [*F*(2,44) = 5.739, *p* = 0.006] (**Figure 4B**). In E2 mice, there was an effect of day [*F*(1,13) = 14.41, *p* = 0.0022] and a trend towards an effect of treatment [*F*(1,13) = 3.407, *p* = 0.0878]. In E3 mice, there was only an effect of day [*F*(1,15) = 25.7, *p* = 0.0001]. Similarly, in E4 mice, there was only an effect of day [*F*(1,17) = 36.75, *p* < 0.0001]. When the activity level in the open field containing objects (days 3 and 4) during week 2 was analyzed, there was an effect of treatment [*F*(1,44) = 4.588, *p* = 0.038] (**Figure 4B**).

When time spent in the center of the open field during week 1 was analyzed, there was an effect of day [*F*(1,44) = 29.993, *p* < 0.001], with less time spent in the center of the open field on day 2 than day 1 (**Figure 4C**). When time spent in the center of the open field containing objects during week 1 was analyzed, there was an effect of sex [*F*(1,43) = 4.463, *p* = 0.040] and an effect of genotype [*F*(2,43) = 4.839, *p* = 0.013], with E2 mice spending more time in the center than E3 (*p* = 0.0155, Tukey’s) and E4 (*p* = 0.0024, Tukey’s) mice (**Figure 4C**).

When time spent in the center of the open field during week 2 was analyzed, there was an effect of sex [*F*(1,43) = 7.839, *p* = 0.008] and a trend towards an effect of genotype [*F*(2,43) = 2.708, *p* = 0.078] (**Figure 4D**). When time spent in the center of the open field containing objects during week 2 was analyzed, there was a day × sex interaction [*F*(1,43) = 4.637, *p* = 0.037], an effect of genotype [*F*(2,43) = 4.477, *p* = 0.017], with E2 mice spending more time in the center than E4 mice (*p* = 0.0333, Tukey’s) and a trend towards spending more time in the center than E3 mice (*p* = 0.0928, Tukey’s), and a trend towards an effect of sex [*F*(1,43) = 3.860, *p* = 0.056].

Next, object recognition was assessed. During week 1, vehicle-treated E2 (*p* = 0.0531, paired t-test) and E3 mice (*p* = 0.0783, paired *t*-test) showed a trend towards exploring the novel object more than the familiar one (**Figure 4E**). In contrast, VLP-treated E2 and E3 mice and vehicle- and VLP-treated E4 mice did not. During week 2, only vehicle-treated E3 mice explored the novel object more than the familiar one (*p* = 0.0176, paired *t*-test) (**Figure 4F**).

### 3.3 Rotarod

**Figure 5:**
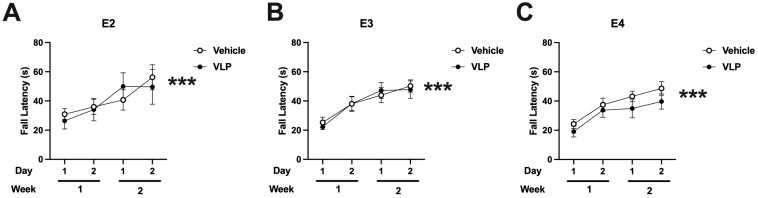
Rotarod performance of vehicle- and VLP-treated E2 **(A)**, E3 **(B)**, and E4 **(C)** mice. All groups improved their performance with training. Effect of day: ***p < 0.001.

When performance on the rotarod was analyzed, there was an effect of day [*F*(1,44) = 62.701, *p* < 0.001] with improved performance with training. There were no effects of genotype or treatment (**Figure 5**).

### 3.4 Grip strength

**Figure 6:**
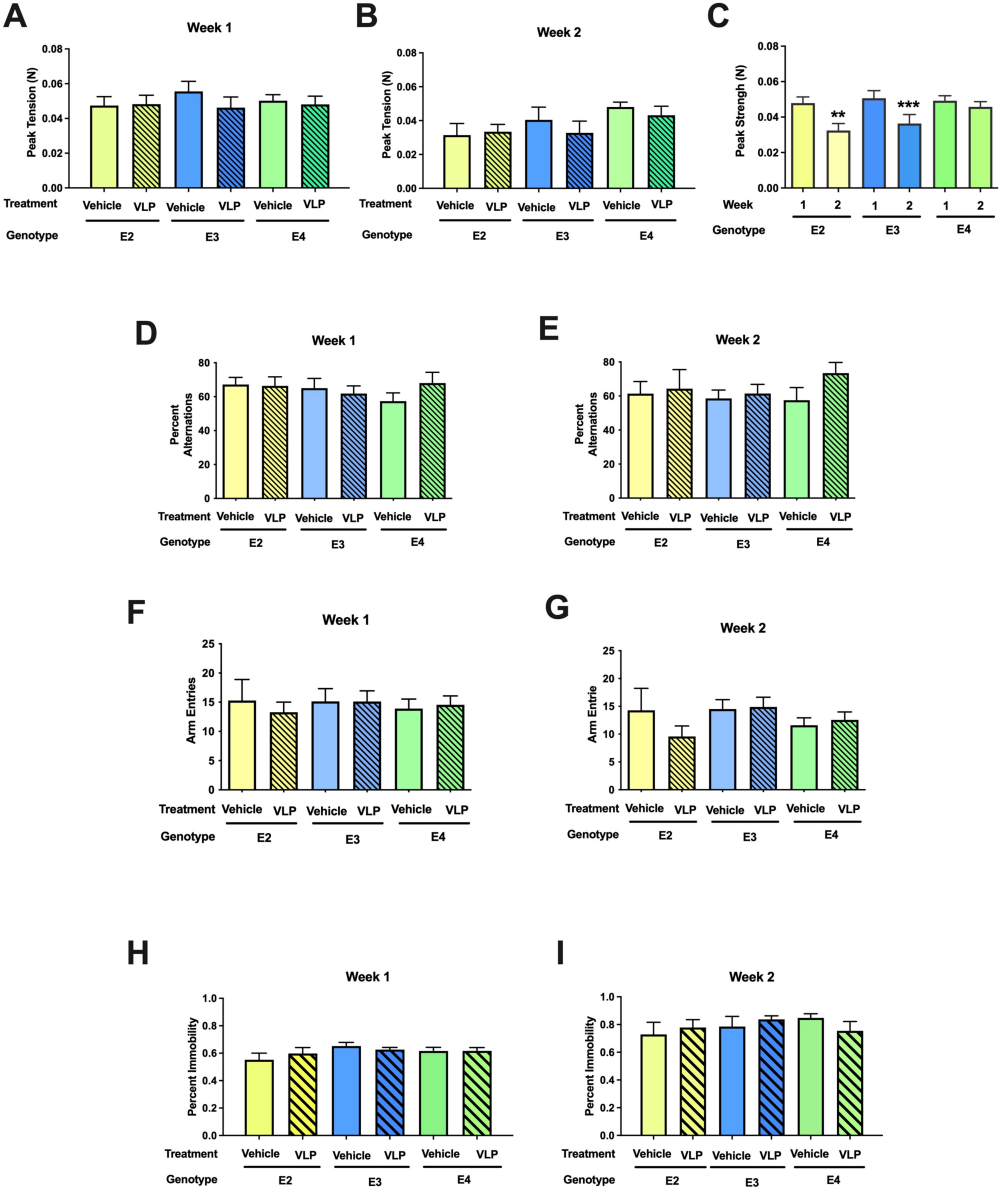
**(A–C)** Behavioral performance of VLP- and vehicle-treated E2, E3, and E4 mice in the grip strength test. There was a week × genotype [*F*(2,44) = 4.747, *p* = 0.014] interaction. In E2 and E3 mice, the grip strength in week 2 was lower than that in week 1 in E2. **p = 0.0036, ***p = 0.0009, paired *t*-tests. This was not seen in E4 mice. **(D, E)** Spontaneous alternation of VLP- and vehicle-treated E2, E3, and E4 mice in the Y maze. **(F, G)** Arm entries of VLP- and vehicle-treated E2, E3, and E4 mice in the Y maze. **(H, I)** Depressive-like behavior of VLP- and vehicle-treated E2, E3, and E4 mice in the forced swim test.

For grip strength, there was a week × sex [*F*(1,44) = 5.25, *p* = 0.027] and a week × genotype [*F*(2,44) = 4.747, *p* = 0.014] interaction. While there was no genotype or treatment effect in week 1 (**Figure 6A**) or week 2 (**Figure 6B**), the grip strength in week 2 was lower than that in week 1 in E2 (*t* = 3.547, *p* = 0.0036, paired t-test) and E3 (*t* = 4.049, *p* = 0.0009, paired *t*-test), but not E4 mice (**Figure 6C**).

### 3.5 Y maze

When spontaneous alternation was assessed in the Y maze, there was only an effect of week [*F*(1,44) = 4.263, *p* = 0.045], with lower spontaneous alternation in week 2 than in week 1 (**Figures 6D, E**). We recognize that this might be due to the larger Y maze used in week 2 than in week 1. When activity levels were analyzed in the Y maze, there was an effect of sex [*F*(1,43) = 6.433, *p* = 0.015] and a trend towards a week × genotype interaction [*F*(2,43) = 3.024, *p* = 0.059] (**Figures 6F, G**).

### 3.6 Forced swim test

When depressive-like behavior was tested in the forced swim test, there was an effect of week [*F*(1,44) = 8.981, *p* = 0.004], with more depressive-like behavior in week 2 than in week 1, but no effect of genotype or treatment (**Figures 6H, I**).

### 3.7 Circadian body temperatures of VLP- and vehicle-treated E2, E3, and E4 mice

**Figure 7:**
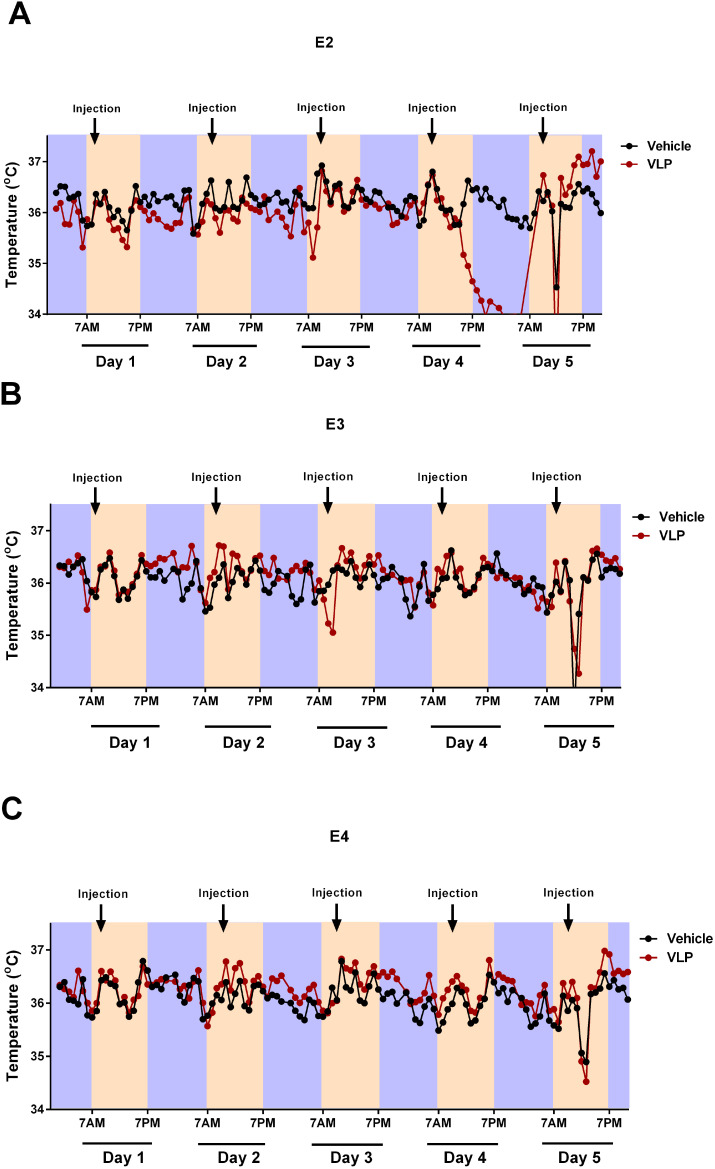
Circadian body temperatures in VLP- and vehicle-treated E2 **(A)**, E3 **(B)**, and E4 **(C)** mice. Black indicates the Vehicle groups and red the VLP groups. The dark periods are indicated in purple, the light periods in peach.

The circadian body temperatures during the first week of behavioral testing are illustrated in **Figure 7**. Based on the pattern of the data observed, the light and dark periods were analyzed as separate analyses, with the mean body temperature in the light or dark period of each day as the repeated measure.

During the light periods, there was only an effect of day [*F*(2.771,110.855) = 22.958, *p* < 0.001, Greenhouse–Geisser correction]. However, during the dark periods, there was an effect of day [*F*(2.495,99.780) = 22.958, *p =* 0.005, Greenhouse–Geisser correction] and a day × genotype × treatment interaction [*F*(4.989,99.780) = 2.518, *p* = 0.034, Greenhouse–Geisser correction], with the E2 mice more affected by VLP treatment than E3 or E4 mice. In general, in E2 mice (**Figure 7A**), body temperatures were lower in VLP- than in vehicle-treated mice, with the most profound effect seen in the dark period on day 4. In contrast, in E3 (**Figure 7B**) and E4 (**Figure 7C**) mice, a more subtle higher body temperature in VLP- than in vehicle-treated mice was seen.

Based on this three-way interaction, we also analyzed each treatment group separately. In the vehicle treatment group, no significant effects or trends were seen. However, in the VLP treatment group, there was a trend towards an effect of day [*F*(3,57) = 2.668, *p* = 0.056] and a trend towards a day × genotype interaction [*F*(6,57) = 2.159, *p* = 0.060].

We also analyzed each light and dark period separately, using the hour as the repeated measure.

During the light period, on day 1, there was only an effect of hour [*F*(5.238,183.317) = 22.958, *p* < 0.001, Greenhouse–Geisser correction]. However, during the light period on day 2, there was an effect of hour [*F*(6.385,197.920) = 22.958, *p* < 0.001, Greenhouse–Geisser correction] and a hour × genotype interaction [*F*(12.769,183.317) = 1.970, *p =* 0.026, Greenhouse–Geisser correction]. During the light period on day 3, there was only an effect of hour [*F*(3.501,108.519) = 8.609, *p* < 0.001, Greenhouse–Geisser correction]. During the light period on day 4, there was an effect of hour [*F*(4.920,142.685) = 13.965, *p* < 0.001, Greenhouse–Geisser correction] and a trend towards an hour × genotype interaction [*F*(9.840,142.685) = 1.832, *p =* 0.061, Greenhouse–Geisser correction]. During the light period on day 5, there was an effect of hour [*F*(2.072,64.239) = 1.832, *p <* 0.001, Greenhouse–Geisser correction] and a trend towards an effect of genotype [*F*(2,31) = 3.120, *p =* 0.058].

During the dark period, on day 1, there was only an effect of hour [*F*(6.971,223.069) = 5.847, *p* < 0.001, Greenhouse–Geisser correction]. During the dark period, on day 2, there was only an effect of hour [*F*(6.323,227.621) = 5.378, *p* < 0.001, Greenhouse–Geisser correction]. During the dark period, on day 3, there was only an effect of hour [*F*(7.293,269.855) = 9.508, *p* < 0.001, Greenhouse–Geisser correction]. During the dark period, on day 4, there was an effect of hour [*F*(5.745,172.353) = 7.596, *p* < 0.001, Greenhouse–Geisser correction].

### 3.8 PCA

Nine factors were identified with eigenvalues < 1.0 and that explained a total of 81.3% of the variance among the behavioral measures (**Table 2**). Distance in the open field on 7 out of 8 days, distance moved in the center of the open field in 6 out of 8 days, entries and spontaneous alternation in the Y maze in week 2, and the body weight ratio in week 2 all loaded on Factor 1, indicating a common underlying ability being assessed by all these behavioral measures. The directions of the component loadings in Factor 1 were such that increasing values of the factor indicate higher activity measures and increased cognitive performance in the Y maze and an increased body weight ratio in week 2.

**Table 2:** Component loadings of behavioral measures in the PCA^1^.

	F1	F2	F3	F4	F5	F6	F7	F8	F9
TDOFD1w1	**0.732**								
TDOFD2w1	**0.799**								
TDOFD3w1	**0.825**								
TDOFD4w1	**0.772**								
TDOFD1w2	**0.844**								
TDOFD2w2	**0.526**		**−0.758**						
TDOFD3w2			**−0.841**						
TDOFD4w2	0.490		**−0.811**						
CDOFD1w1						**0.561**	0.451		
CDOFD2w1						0.480			
CDOFD3w1	**0.597**	**−**0.406							
CDOFD4w1	**0.685**	**−**0.470							
CDOFD1w2	**0.525**	**−0.601**							
CDOFD2w2	**0.514**	**−0.595**							
CDOFD3w2	0.434	**−0.733**							
CDOFD3w2	**0.620**	**−**0.481							
DIw1									
DIw2							**0.632**		**0.501**
Immobility FSTw1								**0.575**	0.414
Immobility FSTw2			**0.684**						
Entries Ymazew1					**0.593**				
SpontAlternationw1					**0.693**				
Entries Ymazew2	**0.516**	**0.509**							
SpontAlternationw2	0.476								
GripStrengthw1					0.470				0.426
GripStrengthw2					0.401			**−0.512**	
RRD1w1		0.474		**0.694**					
RRD2w1		0.481		**0.556**					
RRD1w2		0.463		**0.766**					
RRD2w2		**0.597**		**0.561**					
BWRatiow1						**0.547**	**−**0.471		
BWRatiow2	0.430					**0.566**	**−**0.401		
Eigenvalues	7.180	4.441	3.599	2.652	2.311	1.999	1.499	1.170	1.157
Percentage of variance explained	16.2	14.5	12.1	9.4	7.8	6.1	6.0	5.4	4.0

^1^Loadings higher than 0.5 are indicated in bold. F indicates the factor.

Distance moved in the center of the open field in 6 out of 8 days, entries in Y maze in week 2, and fall latency on all 4 days in the rotarod test all loaded on Factor 2. The directions of the component loading in Factor 2 were such that reduced activity levels in the center of the open field and in week 2 and increased activity levels in the Y maze in week 2 indicate increased sensorimotor performance in the rotarod test.

Distance in the open field in 3 out of 4 days in the open field in week 2 and percent immobility in the forced swim test in week 2 loaded on Factor 3. The directions of the component loading in Factor 3 were such that decreased activity in the open field in week 2 indicates increased depressive-like behavior in the forced swim test in week 2.

Performance on all day of the rotarod test exclusively loaded on Factor 4.

Entries and spontaneous alternation in the Y maze in week 1 and grip strengths in weeks 1 and 2 loaded on Factor 5. The direction of the component loading was such that increased activity levels and increased cognitive performance in the Y maze in week 1 indicate increased grip strength in both weeks.

Activity levels in the center of the open field days 1 and 2 in week 1 and body weight ratios in both weeks loaded on Factor 6. The direction of the component loading was such that increased activity levels in the center of the open field in week 1 indicate increased body ratios in both weeks.

The activity levels in the center of the open field on day 1 of open field testing in week 1, the discrimination index in the object recognition test in week 2, and body weight ratios in both weeks loaded on Factor 7. The direction of the component loading was such that increased activity in the center of the open field in the first day of open field testing in week 1 indicate better cognitive performance in the object recognition test but lower body weight ratios in both weeks.

Depressive-like behavior in the forced swim test in week 1 and grip strength in week 2 loaded on Factor 8. The direction of the component loading was such that increased depressive-like behavior in the forced swim test indicate reduced grip strength in week 2.

The discrimination index in the object recognition test in week 2, depressive-like behavior in the forced swim test in week 1, and grip strength in week 1 loaded on Factor 9. The direction of the component loadings was such that more depressive-like behavior in the forced swim test in week 1 and more grip strength in week 1 indicate better cognitive performance in the object recognition test in week 2.

### 3.9 Hippocampal cytokine expression of VLP- and vehicle-treated E2, E3, and E4 mice

**Figure 8:**
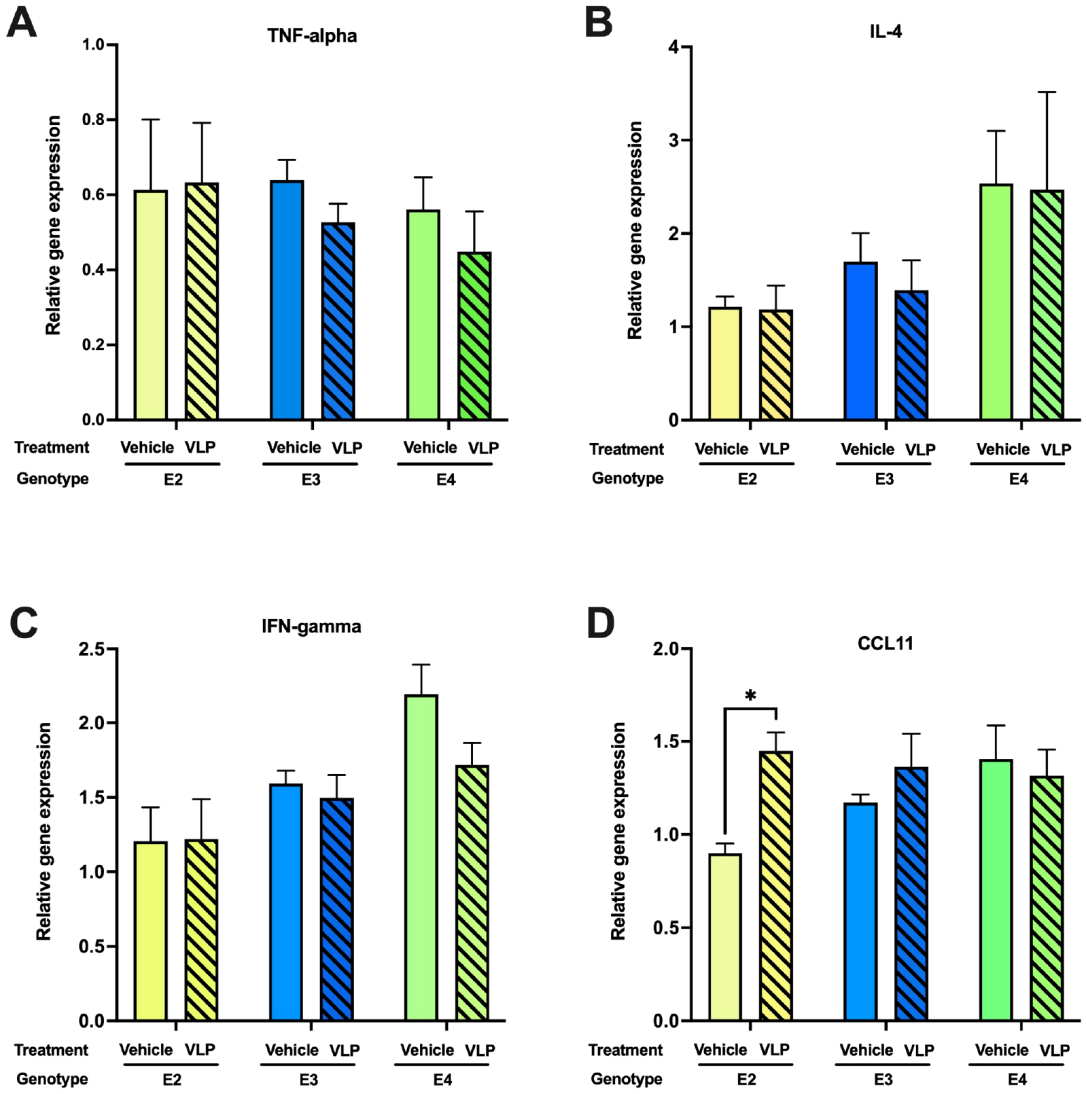
Hippocampal cytokine expression in VLP- and vehicle-treated mice. In E2 mice, **(A)** There were no genotype or treatment differences in TNF-alpha expression levels. **(B)** There were no genotype or treatment differences in IL-4 expression levels. **(C)** There were no genotype or treatment differences in IFN-gamma expression levels. **(D)** CCL11 mRNA expression was higher in VLP- than in vehicle-treated mice. *p < 0.05, *t*-test. *n* = 3–5/genotype/treatment.

Acute SARS-CoV-2 pathology is largely driven by inflammatory factors that mediate damage to the lungs. Notably, longer-term cognitive sequelae (“brain fog”) are also associated with dysregulated cytokine expression in serum and CSF (24). We examined mRNA expression of several inflammatory cytokines in the hippocampi of VLP/saline-treated mice. Mice were euthanized after the last behavioral test (on the day of the last VLP injection). RNA isolated from hippocampi was analyzed by qRT-PCR for expression of the inflammatory mediators TNF-α, IFN-γ, and IL-4. We also analyzed expression of CCL11, which has been implicated in cognitive dysfunction post-COVID in experimentally infected mice and human patients (24) (**Figure 8**). Although no significant changes were observed for TNF-α, IFN-γ, and IL-4, CCL11 expression was elevated in VLP-treated animals of the E2 genotype. Thus, expression of TNF-α, which was reported to be induced in the brains of SARS-CoV-2-infected mice at 7 dpi, but not at 7 weeks (24), was not significantly changed in any genotype by VLP treatment.

## 4 Discussion

A summary of the effects of VLPs and trends towards effects of VLPs is indicated in **Table 3**. The genotype × treatment interaction seen for activity levels in the open field containing objects in week 2 and the day × genotype × treatment interaction observed for circadian body temperatures in week 1 indicate that for some behavioral measures, the effects of VLPs are apoE isoform-dependent, with the E2 mice being more affected than E3 or E4 mice. The overall decreased activity in the open field containing objects in week 2 indicates that VLPs can also reduce activity levels in an apoE isoform-independent fashion. The fact that effects of VLPs on activity levels in the open field in the absence and presence of objects were seen in week 2 but not in week 1 suggests that these effects might have been due to cumulative effects of VLPs, including administration to VLPs in week 1. The day × genotype × treatment interaction observed for circadian body temperature in week 1 is also consistent with the effects of cumulative exposure; the most pronounced effects of VLPs on body temperature in E2 mice were seen during the dark period on day 4, following the fourth administration of VLPs. The reduced body temperature in E2 mice is consistent with the reduced body temperature of K18-hACE2 transgenic mice that showed more severe disease, weight loss, decreased body temperature, and increased mortality following infection with a low dose of SARS-CoV-2 (32).

**Table 3:** Summary of effects and trends towards effects of VLPs^1^.

Measure	E2	E3	E4	Overall
Activity levels in the open field without objects in week 2	Trend of a treatment effect: VLP < Vehicle			Genotype and genotype × treatment interaction
Activity levels in the open field with objects in week 2				Effect of treatment: VLP < Vehicle
Object recognition in week 2		Impaired object recognition in VLP-treated mice		
Circadian body temperatures during the dark period in week 1	VLP < Vehicle, most pronounced in the dark period of day 4	VLP > Vehicle	VLP > Vehicle	A day × genotype × treatment interaction: E2 mice more affected by VLPs than E3 or E4 mice

^1^The significant effects are indicated in orange.

Compared to vehicle treatment, VLP treatment reduced activity levels in the open field containing objects in week 2. The directions of the component loadings in Factor 1 of the PCA were such that higher activity measures in both weeks indicate increased cognitive performance in the Y maze and an increased body weight ratio in week 2. The directions of the component loading in Factor 3 were such that decreased activity in the open field in week 2 indicate increased depressive-like behavior in the forced swim test in week 2. The direction of the component loading for Factor 5 was such that increased activity levels and increased cognitive performance in the Y maze in week 1 indicate increased grip strength in both weeks. The direction of the component loading for Factor 6 was such that increased activity levels in the center of the open field in week 1 indicate increased body ratios in both weeks. These data indicate that reduced activity levels might contribute to reduced cognitive performance, increased depressive-like behavior, and a reduced body weight ratio. This pattern is consistent with the beneficial effects of activity on cognitive performance (33) and reduced depression (34).

ApoE is involved in the pathogenesis and susceptibility to other infectious diseases, including herpes simplex virus-1, hepatitis C virus, hepatitis E virus, varicella zoster virus, Epstein–Barr virus, malaria, *L. monocytogenes* (LM), and *K. pneumoniae* (2). Compared to E3, E4 is proposed to be a risk factor for COVID-19 and other viruses. E4 modifies the associations of the angiotensin-converting enzyme (ACE) polymorphisms with neuropsychiatric syndromes in AD (4). E4 is also associated with enhanced entry of human immunodeficiency virus 1 (HIV-1) cell entry and HIV-1 disease progression (1). ApoE is an HIV-1-inducible inhibitor of viral production and infectivity in macrophages (2). Consistent with this pattern, the *APOE* genotype was associated with survival in patients infected with COVID-19 and part of the UK Biobank (5); E4 homozygote carriers showed poorer survival than E3 homozygote carriers. A trend towards lower survival of E2 homozygote carriers than E3 homozygote carriers was seen, but this did not reach significance. In a Finish Biobank study, E4 was associated with severe COVID-19 with more prevalent microhemorrhages in intensive care patients (6). It should be noted that in this study potential effects of E2 were not assessed.

Consistent with the human studies, in a mouse model of herpes simplex virus 1 (HSV-1), the cerebral load of latent HSV-1 genomic copies, which is associated with the reactivation risk (35), was 10-fold higher in E4 than E3 TR mice (36). In human apoE TR mice infected with mouse-adaptive COVID-19, both E2 and E4 mice showed a faster disease progression, increased viral loads and suppressive adaptive immune responses earlier after infection, and poorer survival than E3 mice. *In vitro*, viral infection was also higher in E2 and E4 than in E3 mice (5). In this study, E4 mice showed the most profound weight loss, with the E2 mice being less affected, as compared to E3 mice (5). In contrast to these viral studies, following VLP treatment, there was a trend towards a week × genotype × treatment interaction in E2, but not in E3 or E4 mice, and there was a trend towards an effect of treatment and a trend towards a lower body weight ratio in VLP- than in vehicle-treated E2 mice. The differences in pattern seen in relative susceptibility following mouse-adaptive viral COVID-19 infection versus following VLPs in E2 and E4 mice, compared to E3 mice, might be due to *APOE* genotype differences in viral replication. While it is an advantage to be able to perform COVID-19-related studies without replicating virus and outside a BSL-3 facility, we recognize that more severe effects and more profound apoE isoform-dependent effects would likely be seen following mouse-adaptive COVID-19 viral inoculation.

In mice most affected by VLPs, E2 mice, hippocampal CCL11 levels were increased. Consistent with these data, long-term cognitive sequelae (“brain fog”) are also associated with increased CCL11 levels in serum and CSF (24). Elevated CCL11 levels are seen in COVID-19 patients (37) and associated with aging and dementia, learning and memory impairments, and reduced neurogenesis (38). Interestingly, elevated CCL11 levels were also seen in a parabiosis study giving young blood to aged mice and *vice versa* and associated with learning and memory impairments when CCL11 was administered to younger mice (39).

Following exposure to COVID-19, men were more likely to be hospitalized, admitted to intensive care units, have a greater inflammatory cytokine production and antiviral antibody levels, and die (40, 41). Consistent with these human data, male Syrian hamsters showed more severe lung injury, a slower recovery, a greater percent body weight loss, and a reduced antibody response following inoculation with SARS-CoV-2/USA-WA1/2020, although viral titers in respiratory tissues and cytokine levels in pulmonary tissues were comparable in males and females (42). In addition, male K18-hACE2 transgenic mice showed more severe disease, weight loss, decreased body temperature, and increased mortality following infection with a low dose of SARS-CoV-2 (32). As no treatment × sex interactions were observed for any outcome measure, female and male data were not analyzed separately. However, a limitation of the current study is that we cannot exclude that increasing the sample of mice might have revealed a treatment × sex interaction. Alternatively, it is conceivable that viral replication is required to detect a treatment × sex interaction. Future efforts are warranted to consider using live-attenuated viral models in the absence and potentially in the presence of VLPs to compare their impacts on behavioral and cognitive performance.

A limitation of the current study design is that mice were behaviorally tested for 2 weeks. It is conceivable that in addition to the daily treatment injections, the behavioral testing itself might have contributed to fatigue in the mice. For example, activity levels in the open field and grip strength were lower in week 2 than in week 1. A study design with treatments for 2 weeks but only behavioral testing in the second week would allow addressing this.

Our VLPs include expression of the SARS-CoV-2 nucleocapsid (N), membrane (M), and envelope (E) structural proteins together with S. The results of the current study indicate that even in the absence of viral replication, detrimental effects on behavioral measures and circadian body temperatures are seen. Efforts are warranted to assess the pathways underlying these effects and to assess whether these effects are long-lasting and might model long COVID in humans. In addition to modeling COVID-19, this model is also relevant to assess the effects of using VLPs as immunization against COVID-19 on the brain. For example, it has recently been hypothesized that the inability to make long-lived plasma cells following COVID-19 vaccination might be related to the larger spacing of S1 molecules than required to bind and fully activate a single B-cell receptor, and there is therefore increased interest in using VLPs for vaccination against COVID-19 (43) (44).

## Data Availability

The original contributions presented in the study are included in the article/supplementary material. Further inquiries can be directed to the corresponding author.

## References

[1] Burt TD Agan BK Marconi VC He W Kulkami H Mold JE. Apolipoprotein (apo) E4 enhances HIV-1 cell entry *in vitro*, and the APOE ϵ4/ϵ4 genotype accelerates HIV disease progression. Proc Nath Acad Sci USA. (2008) 105:8718–23. doi: 10.1073/pnas.0803526105 PMC2438419 18562290

[2] Siddiqui R Suzu S Ueno M Nasser H Koba R Bhuyan F. Apolipoprotein E is an HIV-1-inducible inhibitor of viral production and infectivity in macrophages. PloS Pathog. (2018) 14:e1007372. doi: 10.1371/journal.ppat.1007372 30496280 PMC6289579

[3] Oudit GY Wang K Viveiros A Kellner MJ Penninger JM. Angiotensin-converting enzyme 2—at the heart of the COVID-19 pandemic. Cell. (2023) 186:906–22. doi: 10.1016/j.cell.2023.01.039 PMC9892333 36787743

[4] de Oliviera FF Almeida SS Almeida Junior GV Bertolucci PHF. APOE haplotypes modify the associations of the ACE insertion/deletion polymorphism with neuropsychiatric symptoms in dementia due to Alzheimer’s disease. Eur J Neurol. (2017) 24S1:422. doi: 10.1080/13546805.2021.1931085

[5] Ostendorf BN Patel MA Bilanovic J Hoffmann H-H Carrasco SE Rice CM. Common human genetic variants of APOE impact murine COVID-19 mortality. Nature. (2022) 611:346–51. doi: 10.1038/s41586-022-05344-2 PMC10957240 36130725

[6] Kurki SN Kanonen J Kaivola K Hokkanen L Myranpaa M Puttonen H. APOE ϵ4 associates with increased risk of severe COVID-19, cerebral microhaemorrhages and post-COVID mental fatigue: a Finnish biobank, autopsy and clinical study. Acta Neuropathol Commun. (2021) 9:199. doi: 10.1186/s40478-021-01302-7 34949230 PMC8696243

[7] Solomou I Nikolaou F Michaelides MP Constantinidou F. Long-term psychological impact of the pandemic COVID-19: Identification of high-risk groups and assessment of precautionary measures five months after the first wave of restrictions was lifted. PloS Glob Public Health. (2024) 4:e0002847. doi: 10.1371/journal.pgph.0002847 38394160 PMC10889631

[8] Bobak L Dorney I Kovacevich A Barnett B Kaelber D. Preexisting psychiatric conditions as risk factors for diagnosed long COVID-19 syndrome within aggregated electronic health record data. Psychomat Med. (2024) 86:132–6. doi: 10.1097/PSY.0000000000001280 PMC11001529 38193771

[9] Taylor K Pearson M Das S Sardell J Chocian K Gardner S. Genetic risk factors for severe and fatigue dominant long COVID and commonalities with ME/CFS identified by combinatorial analysis. J Trans Med. (2023) 21:775. doi: 10.1186/s12967-023-04588-4 PMC10621206 37915075

[10] Pszczolowska M Walczak K Miskow W Antosz K Batko J Karska J. Molecular cross-talk between long COVID-19 and Alzheimer’s disease. GeroScience. (2024) 46:2885–99. doi: 10.1007/s11357-024-101096 PMC11009207 38393535

[11] Horsburgh K Graham DI Stewart J Nicoll JAR. Influence of apolipoprotein E genotype on neuronal damage and ApoE immunoreactivity in human hippocampus following global ischemia. J Neuropathol Exp Neurol. (1999) 58:227–34. doi: 10.1097/00005072-199903000-00002 10197814

[12] Raber J Huang Y Ashford JW. ApoE genotype accounts for the vast majority of AD risk and AD pathology. Neurobiol Aging. (2004) 25:641–50. doi: 10.1016/j.neurobiolaging.2003.12.023 15172743

[13] Chartier–Harlin M Parfitt M Legrain S Perez–Tur J Brousseau T Evans A. Apolipoprotein E, ϵ4 allele as a major risk factor for sporadic early- and late-onset forms of Alzheimer’s disease: Analysis of the 19q13.2 chromosomal region. Hum Mol Genet. (1994) 3:569–74. doi: 10.1093/hmg/3.4.569 8069300

[14] Katzman R. Apolipoprotein E and alzheimer’s disease. Curr Opin Neurobiol. (1994) 4:703–7. doi: 10.1016/0959-4388(94)90013-2 7849527

[15] Farrer LA Cupples LA Haines JL Hyman B Kukull WA Mayeux R. Effects of age, sex, and ethnicity on the association between apolipoprotein E genotype and Alzheimer disease. A meta-analysis. J Am Med Assoc. (1997) 278:1349–56. doi: 10.1001/jama.1997.03550160069041 9343467

[16] Ciurleo GCV Tavares-Junior JW Vieira CMAG Braga-Neto P Oria RB. Do APOE4 and long COVID-19 increase the risk for neurodegenerative diseases in adverse environments and poverty? Front Neurosci. (2023) 17:1229073. doi: 10.3389/fnins.2023.1229073 37694114 PMC10483995

[17] Narayanan SA Jamison DA Guarnieri JW Zaksas V Topper M Koutnik AP. A comprehensive SARS-CoV-2 and COVID-19 review, Part 2: host extracellular to systemic effects of SARS-CoV-2 infection. Eur J Hum Genet. (2023) 32:10–20. doi: 10.1038/s41431-023-01462-1 37938797 PMC10772081

[18] Almazan F DeDiego ML Sola I Zuniga S Nieto-Tones JL Marquez-Jurado S. Engineering a replication-competent, propagation-defective Middle East respiratory syndrome coronavirus as a vaccine candidate. MBio. (2013) 4:e00650–13. doi: 10.1128/mBio.00650-13 PMC3774192 24023385

[19] Bisht H Roberts A Vogel L Moss B. Severe acute respiratory syndrome coronavirus spike protein expressed by attenuated vaccinia virus protectively immunizes mice. Proc Nath Acad Sci USA. (2004) 101:6641–6. doi: 10.1073/pnas.0401939101 PMC404098 15096611

[20] Tay TL Savage JC Hui CW Bisht K Tremblay ME. Microglia across the lifespan: from origin to function in brain development, plasticity and cognition. J Physiol. (2017) 595:1929–45. doi: 10.1113/tjp.2017.595.issue-6 PMC5350449 27104646

[21] Sullivan PM Mezdour H Quarfordt SH Maeda N. Type III hyperlipoproteinemia and spontaneous atherosclerosis in mice resulting from gene replacement of mouse Apoe with human Apoe*2. J Clin Invest. (1998) 102:130–5. doi: 10.1172/JCI2673 PMC509074 9649566

[22] Sullivan PM Mezdour H Aratani Y Knouff C Najib J Reddick RL. Targeted replacement of the mouse apolipoprotein E gene with the common human *APOE3* allele enhances diet-induced hypercholesterolemia and atherosclerosis. J Biol Chem. (1997) 272:17972–80. doi: 10.1074/jbc.272.29.17972 9218423

[23] Knouff C Hinsdale ME Mezdour H Altenburg MK Watanabe M Quarfordt SH. ApoE structure determines VLDL clearance and atherosclerosis in mice. J Clin Invest. (1999) 103:1579–86. doi: 10.1172/JCI6172 PMC408371 10359567

[24] Fernandez-Castaneda A Lu P Geraghty AC Song E Lee M-H Wood J. Mild respiratory COVID can cause multi-lineage neural cell and myelin dysregulation. Cell. (2022) 185:2452–68. doi: 10.1016/j.cell.2022.06.008 PMC9189143 35768006

[25] Matuszewski M Afolabi AA Ilesanmi OS Pruc M. Associations between Interleukin-4 and COVID-19 severity: A systematic review and meta-analysis. J Health Soc Sci. (2022) 7:381–96.

[26] Hilligan KL Namasivayam S Clancy CS Baker PJ Old SI Peluf V. Bacterial-induced or passively administered interferon gamma conditions the lung for early control of SARS-CoV-2. Nat Commun. (2023) 14:8229. doi: 10.1038/s41467-023-43447-0 38086794 PMC10716133

[27] Hilligan KL Narmasivayam S Sher A. BCG mediated protection of the lung against experimental SARS-CoV-2 infection. Front Immunol. (2023) 14:1232764. doi: 10.3389/fimmu.2023.1232764 37744331 PMC10514903

[28] Takeshita H Yamamoto K Nozato S Inagaki T Tsuchimochi H Shirai M. [amp]]ldquo;Modified forelimb grip strength test detects aging-associated physiological decline in skeletal muscle function in male mice. Sci Rep. (2017) 7:42323. doi: 10.1038/srep42323 28176863 PMC5296723

[29] Seok Son J Kim H-J Son Y Lee H Chae SAH Seong JK. Effects of exercise-induced apelin levels on skeletal muscle and their capillarization in type 2 diabetic rats. Muscle Nerve. (2017) 56:1155–63. 10.1002/mus.25596 28164323

[30] Taylor TN Caudle WM Shepherd KR Noorian A Jackson CR Iuvone PM. Nonmotor symptoms of Parkinson’s disease revealed in an animal model with reduced monoamine storage capacity. J Neurosci. (2009) 29:8103–13. doi: 10.1523/JNEUROSCI.1495-09.2009 PMC2813143 19553450

[31] Livak KJ Schmittgen TD. Analysis of relative gene expression data using real-time quantitative PCR and the 2(-Delta Delta C(T)) Method. Methods. (2001) 25:402–8. doi: 10.1006/meth.2001.1262 11846609

[32] Aiolfi R Deguchi H Fernandez JF Ahmed J Heuer-Jense M de la torre JC. Sex-specific differences in the pathogenesis, endothelial dysfunction, and hypercoagulability of sars-cov-2 infection in K18-hACE2 mice. Blood. (2022) 140:1674–5. doi: 10.1182/blood-2022-163863

[33] Klimova B Dostalova R. The impact of physical activities on cognitive performance among healthy older individuals. Brain Sci. (2020) 10:377. doi: 10.3390/brainsci10060377 32560126 PMC7349640

[34] Kandola A Lewis G Osborn DPJ Stubbs B Hayes JF. Depressive symptoms and objectively measured physical activity and sedentary behaviour throughout adolescence: a prospective cohort study. Lancet Psych. (2020) 7:262–71. doi: 10.1016/S2215-0366(20)30034-1 PMC7033559 32059797

[35] Hoshino Y Pe’snicak L Cohen JI Straus SE. Rates of reactivation of latent herpes simplex virus from mouse trigeminal ganglia ex vivo correlate directly with viral load and inversely with number of infiltrating CD8+ T cells. J Virol. (2007) 81:8157–64. doi: 10.1128/JVI.00474-07 PMC1951330 17522198

[36] Burgos JS Ramirez C Sastre I Valdivieso F. Effect of apolipoprotein E on the cerebral load of latent herpes simplex virus type 1 DNA. J Virol. (2006) 80:5383–7. doi: 10.1128/JVI.00006-06 PMC1472141 16699018

[37] Nazarinia D Behzadifard M Gholampour J Karimi R Gholampour M. Eotaxin-1 (CCL11) in neuroinflammatory disorders and possible role in COVID-19 neurologic complications. Acta Neurobiol Belg. (2022) 122:865–9. doi: 10.1007/s13760-022-01984-3 PMC9188656 35690992

[38] Lavandoshki P Pierdona V Maurmann RM Grun LK Guma FTCR Barbe-Tuana FM. Eotaxin-1/CCL11 promotes cellular senescence in human-derived fibroblasts through pro-oxidant and pro-inflammatory pathways. Front Immunol. (2023) 14:1243537. doi: 10.3389/fimmu.2023.1243537 37860000 PMC10582634

[39] Villeda SA Luo J Mosher KI Zou B Britschgi M Bier IG. The ageing systemic milieu negatively regulates neurogenesis and cognitive function. Nature. (2011) 477:90–4. doi: 10.1038/nature10357 PMC3170097 21886162

[40] Peckham H. Male sex identified by global COVID-19 meta-analysis as a risk factor for death and ITU admission. Nat Commun. (2020) 11:6317. doi: 10.1038/s41467-020-19741-6 33298944 PMC7726563

[41] Klein SL Pekosz A Park H-S Ursin RL Shapiro JR Benner SE. Sex, age, and hospitalization drive antibody responses in a COVID-19 convalescent plasma donor population. J Clin Invest. (2020) 130:6141–50. doi: 10.1172/JCI142004 PMC7598041 32764200

[42] Dhakai S Ruiz-Bedoya CA Zhou R Creisher PS Villano JS Littlefield K. Sex differences in lung imaging and SARS-coV-2 antibody responses in a COVID-19 golden Syrian hamster model. mBio. (2021) 12:e00974–21. doi: 10.1128/mbio.00974-21 PMC8406232 34253053

[43] Cohen J. Missing immune cells may explain why COVID-19 vaccine protection quickly wanes. Science. (2024) 386. doi: 10.1126/science.zz6to68 39418356

[44] Nguyen DC. SARS-CoV-2-specific plasma cells are not durably established in the bone marrow long-lived compartment after mRNA vaccination. Nat Med. (2024). doi: 10.1038/s41591-024-03278-y PMC11750719 39333316

